# A Novel Nanobody Directed against Ovine Myostatin to Enhance Muscle Growth in Mouse

**DOI:** 10.3390/ani10081398

**Published:** 2020-08-11

**Authors:** Kepeng Ou, Youjian Li, Peng Wu, Jixing Guo, Xiujing Hao, Jinliang Sheng, Chuangfu Chen

**Affiliations:** 1 National and Local Joint Engineering Research Center of Targeted and Innovative Therapeutics, Chongqing Engineering Laboratory of Targeted and Innovative Therapeutics, Chongqing Key Laboratory of Kinase Modulators as Innovative Medicine, Chongqing Collaborative Innovation Center of Targeted and Innovative Therapeutics, College of Pharmacy & International Academy of Targeted Therapeutics and Innovation, Chongqing University of Arts and Sciences, Chongqing 402160, China; kepeng.ou@cqwu.edu.cn (K.O.); liyoujian1228@yahoo.com (Y.L.); 2 College of Animal Sciences, Shihezi University, Shihezi, Xinjiang 832000, China; wpshzu@126.com; 3 College of Life Sciences, Shihezi University, Shihezi, Xinjiang 832000, China; guojixinok@126.com; 4 College of Natural Science, NingXia University, YinChua 750000, China; shuaxinxianlu@163.com

**Keywords:** myostatin, nanobody, recombinant myostatin nanobody, muscle growth

## Abstract

**Simple Summary:**

Myostatin (MSTN) is a negative regulator of myogenesis, and various strategies have been used to improve livestock production by inhibiting MSTN. In this study, we developed a single-variable domain of the heavy chain antibody-recombinant MSTN nanobody (RMN) against MSTN. The selected RMN was expressed, purified, and assessed for its cytotoxicity, affinity, specificity, and the ability to inhibit MSTN. The results demonstrated that RMN could specifically detect and bind MSTN, further inhibit myostatin activity, as well as enhance muscle growth in mice.

**Abstract:**

Myostatin (MSTN) is a member of the transforming growth factor beta superfamily and is a negative regulator of myogenesis. It has been shown to function by controlling the proliferation of myoblasts. MSTN inhibition is considered as a promising treatment for promoting animal growth in livestock. Nanobodies, a special antibody discovered in camel, have arisen as an alternative to conventional antibodies and have shown great potential when used as tools in different biotechnology fields, such as diagnostics and therapy. In this study, we examined the effect of MSTN inhibition by RMN on the muscle growth of mice. The results showed that RMN could specifically detect and bind MSTN, as well as inhibit MSTN activity. A significant increase in skeletal muscle mass was observed after intramuscular injection of RMN into mice. Enhanced muscle growth occurred because of myofiber hypertrophy. These results offer a promising approach to enhance muscle growth that warrants further investigation in domestic animals.

## 1. Introduction

Myostatin (MSTN), a member of the transforming growth factor beta (TGF-β) superfamily, is a well-known negative regulator of skeletal muscle development and growth [1]. Natural mutations of the MSTN gene cause a double-muscling phenotype in various animals, such as cattle [2], sheep [3], dogs [4] and even humans [5]. Moreover, MSTN gene knockout mice displayed a double-muscled phenotype, and an increase in overall muscle mass due to muscle fiber hyperplasia and hypertrophy [6]. These data have suggested that inhibiting MSTN activity might promote muscle growth [7,8]. Up to date, several kinds of molecular strategies have been utilized to improve meat production in livestock animals, for example, using RNA interference or gene knockout to obtain transgenic sheep and making antibodies against MSTN in mice and sheep [9,10,11,12]. However, these approaches have different disadvantages in the current studies, such as high cost, complex operation, and impracticality for widespread applications, as well as concerning the quality of meat of genetically modified livestock. Therefore, it is worth exploring an easier method to operate with higher efficiency and more safe strategy to improve the production of livestock by regulating MSTN. 

Antibodies have been used in medical and research applications on a large scale since the development of efficient antibody production techniques [13]. Antibodies are ideal molecules to be used as targeting reagents because of the capability of affinity and specificity [14]. Conventional antibodies consist of two heavy chains and two light chains. However, Hamers-Casterman et al. first discovered that camelids produced functional antibodies devoid of light chains, which are called single domain antibodies, also known as nanobodies [15]. A nanobody has the common characteristics of a conventional antibody, which is also suitable for research, diagnostics, and therapeutic applications of diseases [16,17]. Moreover, due to the particular properties of nanobodies, including small size, robust structure, simple production, high affinity, and specificity, nanobodies have become an ideal research tool for the development of sophisticated nanobiotechnologies [18]. Meanwhile, building on their unique properties, several laboratories have demonstrated that nanobodies are highly useful reagents for examining dynamic biological systems; for instance, nanobodies have been used to crystallize flexible membrane proteins [19]. 

In this study, we investigated whether administration of recombinant MSTN nanobody (RMN) would inhibit MSTN and promote muscle growth. Using in vitro platforms, we assessed the specific activity of RMN. Additionally, we evaluated the toxicity of RMN in fibroblast cells. Finally, we established an in vivo model to explore the effect of RMN on MSTN inhibition and the muscle performance of mice.

## 2. Materials and Methods 

### 2.1. SDS-PAGE Analysis

The RMN, recombinant MSTN, and BVDV protein were constructed in our previous study [20,21]. For Western blot analysis of specificity of RMN, the equal protein of recombinant MSTN and BVDV protein (set as negative control) were mixed with 4× SDS sample buffer separately, then, boiled for 10 min at 90°C, and resolved using 4% to 20% NuPAGE gels (Invitrogen). Proteins were transferred to PVDF membranes (Invitrogen), blocked with 5% skimmed milk for 2 h, rinsed, and incubated overnight at 4°C with RMN (17 μg/mL). Excess antibody was then removed by washing the membrane in TBS/0.1% Tween 20, and the membranes were incubated for 2 h with horseradish peroxidase-conjugated goat anti-Llama IgG antibody (ab112786, Abcam 1:2000). Following further washes in TBS/0.1% Tween 20, protein signals were developed with an ECL reagent (Sigma, Shanghai, China) and captured by an electronic imaging system (Konica Minolta, Shanghai, China). 

### 2.2. ELISA

A ninety-six-well ELISA plate was coated with recombinant MSTN and BVDV protein overnight at 4 °C. Blocking was performed by applying 100 µL of blocking buffer followed by incubation at 37 °C for 1 h. Then, RMN (1.7 μg/mL, 100 μL) was added and incubated at 37 °C for 1 h. The solution was thoroughly aspirated and the plate was washed 3 times. Next, 100 μL of HRP-conjugated goat anti-Llama IgG antibody (Abcam, Shanghai, China) was added and incubated at 37 °C for 1 h. Following further washes in 0.1% TBST, 100 µL of chromogenic substrate was added into each well and the plate was placed in the dark for 30 min. The reaction was terminated after 30 min and the light absorption of each group was measured at OD 450 nm using a microplate reader.

### 2.3. Cell Culture and Viability

C2C12 myoblasts were cultured in growth medium (DMEM containing 10% FBS and 0.5% gentamycin/ampicillin) in 5% CO_2_ at 37 °C. We used the MTT assay to assess the toxicity of RMN and cell proliferation. C2C12 myoblasts were seeded 24 h prior to MTT at a density of 7.5 × 10^4^ cells/mL in a 96-well plate, then, the medium was removed and replaced with 100 µL of fresh culture medium. To each well, 10 µL of the 12 mM MTT stock solution was added. The negative control of 10 µL of the MTT stock solution was added to 100 µL of medium alone. The cells were incubated at 37 °C for another 4 h. One hundred microlitters of the SDS-HCl solution was added to each well and mixed thoroughly using the pipette before being incubated at 37 °C for 4 h in a humidified chamber. Each sample was mixed again using a pipette and absorbance was read at 570 nm.

### 2.4. Animal Experiments

All experimental procedures and animal care were performed in compliance with the China Code of Practice for the Care and Use of Animals for Scientific Purposes, and conducted under the regulation of the Shihezi University Animals (Scientific Procedures) Act and were in compliance with the use of animals in medical research. Adult (4 weeks old) Balb/c male mice were purchased from the company and randomly divided into two groups (control and treatment), with 6 mice in each group. The mice in the treatment group were injected with RMN in their right (3 mice) or left (3 mice) hind legs (at a dose of 50 μg, 2 μl), and the mice in the control group were injected with the same amount of PBS in their right (3 mice) or left (3 mice) hind legs. All the mice were injected and weighed every other day. After 7 times of injection (from day 1 to day 15), mice were sacrificed by cervical dislocation, and the body and hind legs were weighed. Then, the legs were fixed in 4% PFA for 24 h and snap-frozen in OCT for immunofluorescence staining. Animal experiments were repeated 3 times individually. 

### 2.5. Immunofluorescence and Hematoxylin–Eosin (H&E) Staining

For immunostaining, 8-μm cryosections were washed in PBS and blocked in 5% normal goat serum, 2% BSA, and 0.3% Triton X-100 in PBS for 30 min at room temperature (RT) followed by incubating with the following primary Abs: rabbit anti-MSTN (ab203076, Abcam, 1:50) overnight at 4°C. The secondary Abs was FITC-conjugated goat anti-rabbit IgG (#4412, Cell Signaling Technology) with 1:200 dilution in 2% BSA in PBS at RT for 2 h in the dark. PI (Vector Laboratories) was used to show nuclei in sections. Sections were mounted in antifading medium. H&E staining also was performed with 8-μm cryosections. Images were captured by a Leica SP5-AOBS confocal laser microscope and processed with ImageJ.

### 2.6. Statistics

Results are presented as means ± standard deviation (SD) from a minimum of three independent experiments. Differences between groups were analyzed by Student’s *t*-test or analysis of variance (ANOVA). All the analyses were performed using GraphPad Prism 6 (GraphPad Software, version 6.01). Significant differences were considered at *p* ≦ 0.05.

## 3. Results

### 3.1. Specific Active Detection of RMN

**Figure 1:**
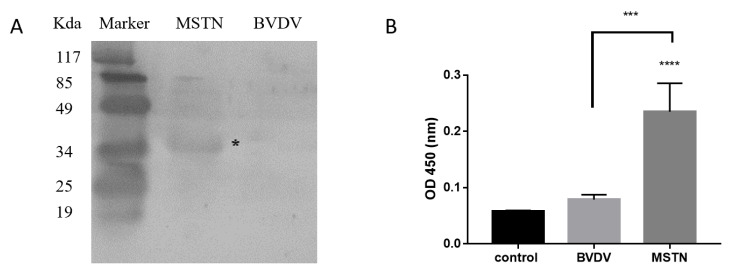
Specific activity of recombinant MSTN nanobody (RMN). (**A**) Western blot showed a specific band at 32 kDa after the recombinant Myostatin (MSTN) protein reacted with RMN (white star). (**B**) The ELISA showed that the RMN was combined with MSTN protein. PBS coating set as control. *** *p* < 0.001; **** *p* < 0.0001, statistical analysis was performed with one-way ANOVA with Dunn’s test for multiple comparison.

In order to assess the specific active detection of RMN, we performed Western blot with the purified RMN. The results showed a specific band at 32 kDa after the recombinant MSTN protein reacted with RMN, while no specific band was detected in the control group (recombinant BVDV protein) (Figure 1A). According to the ELISA detection results, after the RMN was combined with MSTN protein, a significant increase was detected compared to the negative control group (Figure 1B). The results showed the high specificity of RMN and the binding activity was relatively high.

### 3.2. Evaluation of RMN

**Figure 2:**
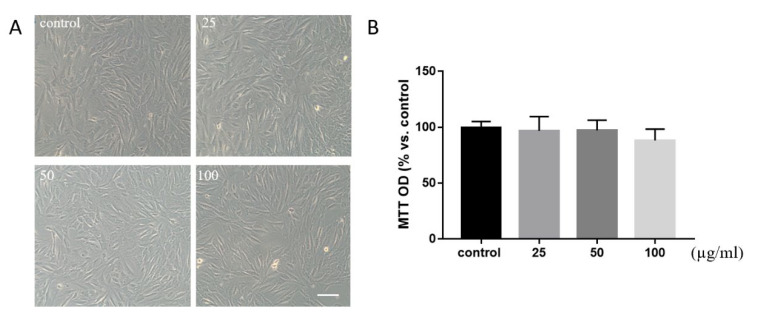
Toxicity of RMN on cells. (**A**) C2C12 myoblast cells showed no difference in growth or quantity compared to the control group from the contrast image. (**B**) The data showed there were not any significant changes after treating by a range concentration of RMN. Scale bar: 100 µm. Statistical analysis was performed with one-way ANOVA with Dunn’s test for multiple comparison.

Due to the highly specific activity of RMN, we therefore wished to interrogate the potential role of RMN in the maintenance and regulation of C2C12 myoblast cells. Using an in vitro approach, we established the response profile of C2C12 following incubation for 24 h with a range of RMN doses of 25, 50, and 100 µg/mL. C2C12 myoblast cells showed no significant difference in growth or quantity compared to the control group from the contrast image (Figure 2A). The data demonstrated that RMN would not affect cell toxicity and proliferation at the dose of 50 μg/mL (Figure 2B).

### 3.3. Effect of RMN Injection on Body and Hind Leg Weight in Mice

**Figure 3:**
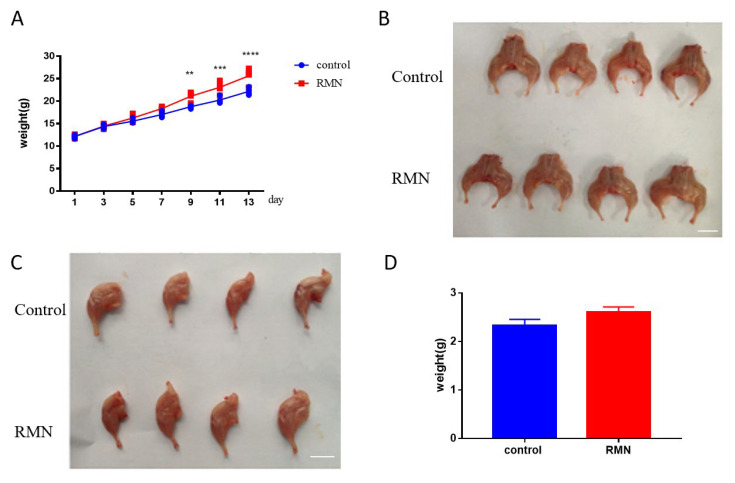
Effect of RMN injection on mouse weight. (**A**,**B**). The total weight significantly increased after the mice were injected four times, and the dissected morphology of the hind leg showed that mice injected with 50 μg RMN in their hind legs presented obviously bigger volume compared to the mice in the PBS group. Scale bar: 1 cm. (**C**,**D**). One side of the hind leg had a bigger diameter compared to the control and there was an increase tendency of weight in treatment with RMN compared to control. Scale bar: 1 cm. *n* = 18. ** *p* < 0.01; *** *p* < 0.001; **** *p* < 0.0001, statistical analysis was performed with Student’s *t*-test for comparison of graph B and graph D.

The in vitro data demonstrated that RMN had highly specific activation and there was no cell toxicity. We next investigated whether this exerted effect on inhibiting muscle grow translated to an in vivo setting. Based on body weight records, the body weight of mice significantly increased after mice were injected four times from 12.16 ± 0.78 g to 18.33 ± 1.31 g (Figure 3A). The dissected morphology of the hind leg showed that mice injected with 50 μg RMN in their hind legs presented obviously bigger compared to the mice in the control group (Figure 3B). One side of muscle tissue was isolated by surgical biopsy; the hind legs of the injection of RMN group were thicker compared to the control (Figure 3C) and there was an increase tendency in weight in treatment group (2.61 ± 0.11 g) compared to control group (2.32 ± 0.13 g) although not significant (Figure 3D).

### 3.4. RMN Increases Muscle Fiber and Inhibits MSTN Protein

**Figure 4:**
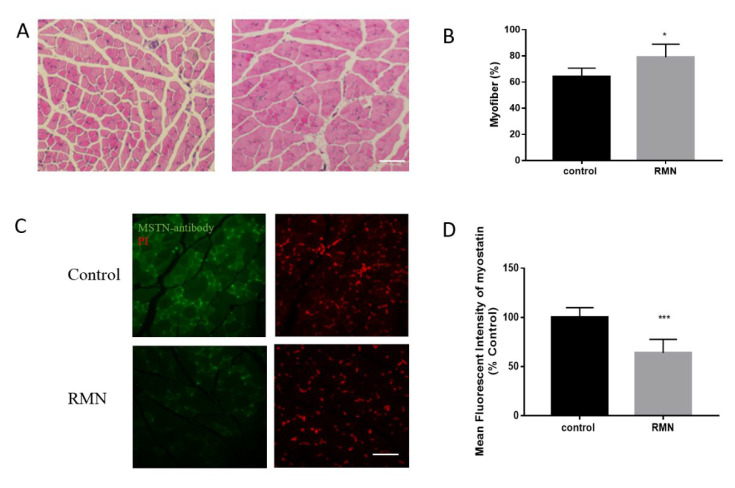
RMN increased muscle fiber and inhibited MSTN protein. (**A**,**B**) H&E staining showed the muscle fiber density of the mice injected with RMN were significantly increased. Scale bar: 100 µm. (**C**,**D**) Tissue immunofluorescence analysis indicated that MSTN protein was expressed in the cytoplasm of the muscle of the mice. The expression of MSTN in the muscle tissue of the mice injected with RMN was distinctly decreased. Scale bar: 50 µm. * *p* < 0.05; *** *p* < 0.001; statistical analysis was performed with Student’s *t*-test for comparison.

To explore the potential effect of RMN on myofiber, mouse hind leg muscle and skeletal muscle morphological analysis was conducted. The H&E staining showed the muscle fiber quantity and density of the mice in the 50 μg RMN treatment group were clearly increased (Figure 4A). The cross-sectional areas of the quadriceps femoris of the mice treated with 50 μg RMN were significantly increased in comparison with PBS injection (Figure 4B). Tissue immunofluorescence analysis indicated that MSTN protein was expressed in the cytoplasm of the muscle of the mice (Figure 4C). However, the expression of MSTN in the muscle tissue of the mice in the 50 μg RMN treatment group was distinctly decreased compared to that of the mice in the PBS control group. Moreover, the expression of MSTN protein was inhibited along with the increase in the cross-sectional area of the muscle fiber (Figure 4D).

## 4. Discussion

This study on myostatin function has raised the possibility that myostatin inhibition may be useful for increasing muscle mass for agricultural applications. In this regard, inhibition of endogenous MSTN has been shown to have beneficial effects on animal growth performance [22,23]. This has shown that MSTN-knockdown transgenic sheep by RNAi and SCNT technology significantly increased the muscle of transgenic sheep [12]. To our knowledge, this is the first report in which RMN has been generated and showed increased muscle phenotype by in vivo muscular injection. Using RMN as an MSTN inhibitor, it might be possible to inhibit animal endogenous MSTN in a much simpler and less expensive manner than other strategies.

MSTN belongs to the TGF-β superfamily and can negatively control muscle growth. Our laboratory has contributed to develop a safe and effective strategy to enhance animal growth and meat production for a long time [11,12,24]. Various studies have shown that inhibiting MSTN activity can effectively promote the growth of animal muscles [25,26]. In previous studies of our group, a shRNA expression cassette was constructed for targeting MSTN and decreased MSTN expression by 90% in sheep fibroblasts [27]. Furthermore, our laboratory constructed a polyclonal antibody against MSTN. In accordance with previous research, the results have shown that conventional antibody is able to inhibit MSTN and increase muscle in mice [28]. Meanwhile, the data demonstrated that antibody had no toxic effect on muscle cells, providing a guarantee for the safety of the MSTN antibody as a heterologous substance. However, the above approaches have different disadvantages. For example, when inhibition of MSTN is by mutant propeptide, which has long-term existence in the body, the immune response and the adverse effects on gonadal function and meat quality need to be further evaluated. In terms of the antibody, the low efficiency and specificity of polyclonal antibody is also a factor that needs to be considered. Therefore, the high effect and specificity of the antibody would be a promising strategy to inhibit MSTN.

Currently, the nanobody is evolving into versatile research and application tool kits for related biomedical and biotechnology applications [18,29,30], including targeting drug delivery and therapy [17], disease diagnosis [31], bioimaging [32], and agricultural and plant protection [33]. Nanobodies are good candidates to improve the physicochemical properties and maintain a degree of recognition when compared to conventional antibodies [34]. The low stability and affinity of antibodies can be overcome by nanobodies due to their high stability and ability to keep affinity parameters in the nanomolar range [35]. In addition, nanobodies can be produced on a large scale with good yields, which can be widely popularized and practical [36,37]. Furthermore, nanobody is still stable and functional at temperatures as high as 80 °C, and is also stable at extreme pH levels. These characteristics would facilitate popularization and utilization. In the current study, it was noted that the total weight of the mice significantly increased after being injected with RMN, but the single hindlimb just showed the trend; the reason might be caused by the experimental mice due to the small change in the total weight of the mice. The effect of RMN will be further evaluated on sheep [16,38].

## 5. Conclusions

In summary, our studies have demonstrated the effectiveness of MSTN inhibition by RMN in mice. Moreover, the low cost, the stability, the possibility of mass production of RMN as well as the ease of storage and operation, allow the development of this strategy to operate not only in livestock production but also to be a potential treatment for human muscle wasting diseases, including sarcopenia and muscular dystrophies. However, owing to the larger animal size and longer growth periods, this strategy still requires further investigation in large animal models.
